# Antibiotics affected the bacterial community structure and diversity in pore water and sediments with cultivated *Phragmites australis* in a typical Chinese shallow lake

**DOI:** 10.3389/fmicb.2023.1155526

**Published:** 2023-03-14

**Authors:** Ling Zhang, Junhong Bai, Yujia Zhai, Kegang Zhang, Zhuoqun Wei, Yaqi Wang, Haizhu Liu, Rong Xiao, Milko A. Jorquera

**Affiliations:** ^1^School of Environment, Beijing Normal University, Beijing, China; ^2^School of Chemistry and Chemical Engineering, Qinghai Normal University, Xining, China; ^3^Department of Environmental Engineering and Science, North China Electric Power University, Baoding, China; ^4^College of Environment and Safety Engineering, FuZhou University, Fuzhou, China; ^5^Laboratorio de Ecología Microbiana Aplicada (EMALAB), Departamento de Ciencias Químicas y Recursos Naturales, Universidad de La Frontera, Temuco, Chile

**Keywords:** pore water, sediments, bacterial community, structure, biodiversity, shallow lake

## Abstract

The migration of antibiotics and bacterial communities between sediments and pore water occurring in the lake, which is affected by aquatic vegetation. However, the differences in bacterial community structure and biodiversity between pore water and sediments with plants in lakes under antibiotic stress are still poorly understood. We collected pore water and sediments in both wild and cultivated *Phragmites australis* regions in the Zaozhadian (ZZD) Lake to explore the characteristics of the bacterial community. Our results showed that the diversity of bacterial community in sediment samples were significantly higher than those in pore water samples in both *P. australis* regions. Due to higher antibiotic levels in sediments from the cultivated *P. australis* region, the composition of bacterial communities showed a difference, which reduced the relative abundance of dominant phyla in pore water and increased that in sediments. The higher bacterial variations in pore water could be explained by sediment in the cultivated *P. australis* region than that in wild *P. australis* region, therefore plant cultivation might change the source-sink pattern between sediments and pore water. The dominant factors shaping the bacterial communities in the wild *P. australis* region pore water or sediment were NH_4_-N, NO_3_-N, and particle size, while cultivated *P. australis* region pore water or sediment were oxytetracycline, tetracycline, etc. The findings of this work indicates that the antibiotic pollution caused by planting activities has a greater impact on the bacterial community, which will provide a reference for the use and management of antibiotics in lake ecosystems.

## 1. Introduction

Lake sediment is one of the main media for the material cycle, including nutrient and pollution transformation and migration. Meanwhile, sediments had high microbial biomass and taxon richness, which plays an important role in driving the biogeochemical cycles of elements (Samuel et al., 2022). However, the aquatic plant growth (Bartelme et al., 2018), and contaminants (such as antibiotics), sedimentation, and chemical gradient between water (or pore water) and sediments (Shade et al., 2012), could lead to the migration of bacterial communities (Zhu et al., 2022a). The toxicological effects (Välitalo et al., 2017) and the pressure selection (Zhu et al., 2019; Sun et al., 2021) of antibiotics on bacterial communities ultimately change the bacteria populations, which could affect the migration of bacterial communities and the balance of the ecosystem. However, the distribution of antibiotics in aquatic environments was different, such as in pore water and sediments (Xu et al., 2014; Zhang et al., 2022a,b). Keshri et al. (2018) found that pore water and sediment share 6.7–20.3% of operational taxonomic units (OTUs), which indicated a link between sediment bacterial communities and pore water bacterial communities. Nevertheless, the origin and differences between bacterial communities in sediments and pore water in lakes affected by antibiotic pollution are still unclear.

Aquatic plants were one of the important components of the lake ecosystem and play an important role in the fate of pollutants and bacterial communities in lakes (Perez-Jaramillo et al., 2018; Zhang et al., 2022a,b). Plants accumulated antibiotics (Zhang et al., 2022a,b) and transfer their metabolites to the bacterial community in the form of root exudates, thereby affecting the changes in the bacterial community (Huang et al., 2020). However, there is evidence that cultivated and wild plants respond differently to environmental stress. Pantigoso et al. (2020) reported that environmental stress has a significant effect on the rhizosphere soil microflora of cultivated and wild potato plants. Besides, the wild and cultivated tomatoes also showed microbial community structural differences caused by soil properties and environmental pressures (Tronson et al., 2022). Similarly, wild and cultivated *P. australis* also show different responses to antibiotics. Compared to wild Phragmites australis (*P. australis*), cultivated *P. australis* had a developed root system, high plant height, and large stem and leaf area, which lead to more antibiotics accumulation from sediments and pore water (Zhang et al., 2022a,b) and bacteria enrichment (Pantigoso et al., 2020). Similarity, the bacterial community has received much influence due to their associations with plant growth and environmental pollution in lake ecosystems (Marschner et al., 2004). While, few studies have involved in the difference in bacterial communities in pore water and sediments covered by wild and cultivated *P. australis*.

Baiyangdian Lake (38°43′ ~ 39°02′N, 115°38′ ~ 116°07′E) is the largest freshwater lake wetland in North China and is located in the Xiong’an New District. Zaozhadian Lake (ZZD, Supplementary Figure S1) is one of the seven large lakes belonging to the Baiyangdian Lake and has a relatively important ecological geographic location. However, ZZD was an important planting area, covering large areas of wild and cultivated *P. australis*. As well as there was occurred antibiotics pollution due to river runoff caused by Pu River inflow and heavy agricultural activities and rural domestic sewage discharge (Cai et al., 2021). Generally, compared with the wild *P. australis* region, total antibiotics in sediments of the cultivated *P. australis* region was higher, while no significant difference was observed in the pore water in our previous study. What is more, wild and cultivated *P. australis* had different antibiotic accumulation abilities (Zhang et al., 2022a,b). However, antibiotics will produce selection pressure on the bacterial community, and the bacterial community affects the growth and development of plants. Therefore, it is necessary to study the difference in bacterial communities in pore water and sediments covered by wild and cultivated *P. australis*, which will have important reference significance for the biological control and management of antibiotic pollution in the lake system.

## 2. Alpha and beta diversity of the bacterial community in the pore water and sediments

Using Illumina 16S rRNA gene sequencing, a total of 396,527 bacterial sequences were detected. 236,822 and 159,705 ASVs were clustered to further compare the bacterial community structure in pore water and sediments, respectively. We evaluated the community richness (Sobs indices), community diversity (Shannon indices), community evenness (Shannoneven indices), and phylogenetic diversity (PD) to further compare the bacterial community structure in pore water and sediment samples. As shown in Supplementary Figures S2A–D, no significant differences in Sobs and PD indices were observed in pore water and sediments in both *P. australis* regions (*p* > 0.05), while the community diversity in sediments (Shannoneven indices: about 0.93, Shannon indices: from 6.52 to 6.59) was significantly (*p* < 0.05) higher than those in pore water. However, there was no significant (*p* > 0.05, Supplementary Figure S3) difference in alpha diversity of the pore water bacterial community between the wild and cultivated *P. australis* regions.

Furthermore, the microbial structure was explored by PCoA and Adonis analysis based on the Bray–Curtis dissimilarity (Supplementary Figures S2E–G). It showed a clear separation in bacterial composition between the pore water and sediment samples in the wild *P. australis* region (*R*^2^ = 0.4277, *p* < 0.05, Supplementary Figure S2F) and in cultivated *P. australis* region (*R*^2^ = 0.4158, *p* < 0.05, Supplementary Figure S2G). However, no significant differences in bacterial community structure in the pore water and sediments from both regions (*p* > 0.05, Supplementary Figure S2E). In the wild *P. australis* region, the PC1 and PC2 axes explained 59.18% of the variation in the bacterial community (Supplementary Figure S2F) and 55.73% in the cultivated *P. australis* region (Supplementary Figure S2G). Along the PC1 axis, the bacterial community in the pore water and sediments was separated (Supplementary Figures S2F,G).

## 3. Composition of the bacterial community in the pore water and sediments

**Figure 1:**
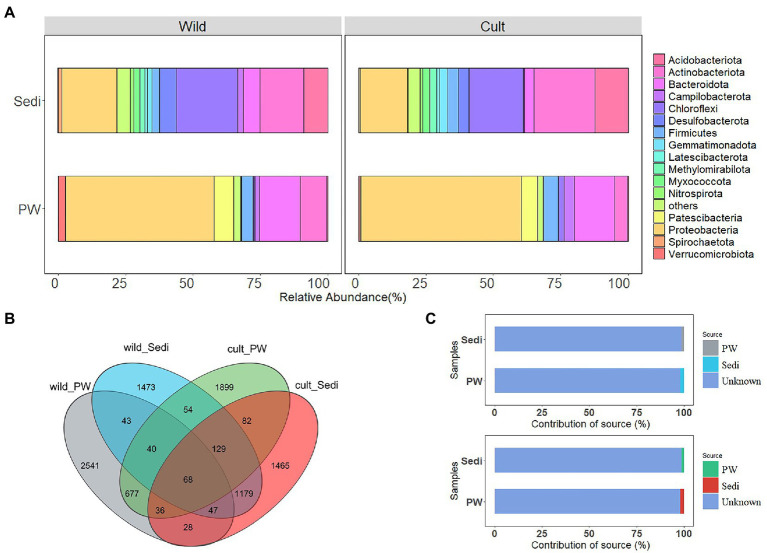
Variations in all bacterial alpha **(A)**, Venn diagram represents the number of ASVs detected **(B)** and contribution of the source to bacterial communities by the Source Tracker **(C)** in the pore water and sediment samples from the wild and cultivated *Phragmites australis* regions.

As shown in Figure 1A, in pore water samples from the wild *P. australis* region, the majority of sequences belonged to Proteobacteria and accounted for 54.97% of the total reads. Bacteroidota for 15.16%, Actinobacteriota accounted for 9.93%, and Patescibacteria for 7.23% of the total reads at the phylum level. However, Chloroflexi (22.74%), Proteobacteria (20.32%), and Actinobacteriota (16.18%), Acidobacteriota (9.00%) were predominant in sediment samples in the wild *P. australis* region. Generally, Proteobacteria (average relative abundance: 59.46%), Bacteroidota (15.01%), and Patescibacteria (5.94%), Firmicutes (5.47%), and Actinobacteriota (4.71%) were identified to be abundant in pore water from the cultivated *P. australis* region. In contrast, the dominant phyla were Actinobacteriota (22.64%), Chloroflexi (20.23%), and Proteobacteria (17.68%), Acidobacteriota (12.36%) in sediment samples in this region (Figure 1A).

Figure 1B illustrated that only 1.07% (43 ASVs) of ASVs were shared between the pore water and sediment samples in the wild *P. australis* region, while 2.44% (84 ASVs) of ASVs were shared in cultivated *P. australis* region. Comparatively, the less ASV number of bacterial communities was observed in sediment samples compared with pore water samples in both *P. australis* regions (Figure 1B). However, when we compare wild and cultivated *P. australis* region, 40.12% (1,179 ASVs) of ASVs were shared in sediment samples, while 15.25% (677 ASVs) of ASVs were shared in pore water.

The possible contributions of bacterial communities in the pore water and sediments to each other were determined using Source Tracker Analysis (Figure 1C). For sediment samples, approximately 1.28% of the variations in bacterial composition could be attributed to the contribution of the pore water in both *P. australis* region. As for pore water, only 1.01% of the bacterial variations in the pore water could be explained by sediment samples in the wild *P. australis* region, while 2.04% in the cultivated *P. australis* region.

**Figure 2:**
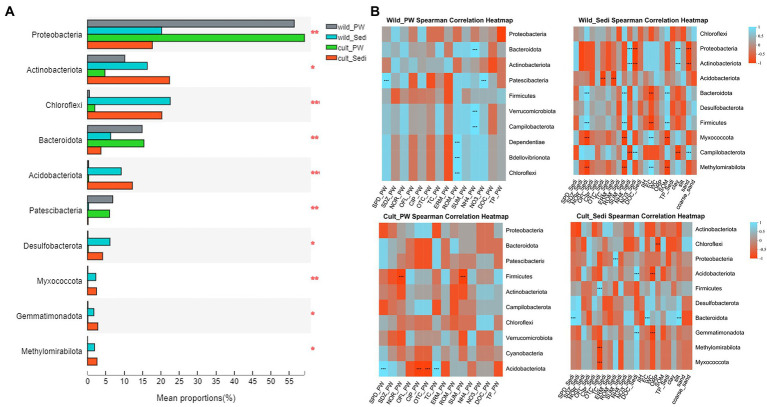
**(A)** One-way ANOVA analysis showed bacterial alpha difference between pore water and sediments in both *Phragmites australis* region and **(B)** spearman correlation between environmental factors and bacteria at the phylum level in the pore water and sediments from the wild and cultivated *P. australis* regions. PW, pore water; Sedi, sediments; SPD, sulfapyridine; SDZ, sulfapyridine; NOR, norfloxacin; OFL, ofloxacin; CIP, ciprofloxacin; OTC, oxytetracycline; TC, tetracycline; ERM, erythromycin; ROM, roxithromycin; and SUM, total antibiotic.

One-way ANOVA results showed significant differences in the relative abundance of Proteobacteria (*p* < 0.01), Actinobacteriota (*p* < 0.05), Chloroflexi (*p* < 0.001), Bacteroidota (*p* < 0.01), and Acidobacteriota (*p* < 0.001) between pore water and sediment samples in wild and cultivated *P. australis* regions, while no significant differences in the relative abundance of Firmicutes were observed between four groups (*p* > 0.05, Figure 2A). However, Proteobacteria and Bacteroidota showed significantly (*p* < 0.05) higher relative abundance in pore water than sediments, while Chloroflexi and Acidobacteriota showed significantly (*p* < 0.05) lower relative abundance in pore water compared with sediments in both *P. australis* region (Supplementary Figure S4).

## 4. Influencing factors on bacterial community in the pore water and sediment

For bacterial communities in the pore water samples in the wild *P. australis* region, the relative abundance of the most dominant phyla Bacteroidota, and Verrucomicrobiota showed significant positive correlation with NH_4_-N (*p* < 0.001), and Patescibacteria demonstrated significant positive correlation with NO_3_-N and sulfapyridine(SPD; *p* < 0.001), respectively (Figure 2B). Similarity, the relative abundance of Proteobacteria and Actinobacteriota in sediment samples in the wild *P. australis* region showed significant positive correlation with NH_4_-N and clay (*p* < 0.001), while significant negative correlation with NO_3_-N and sand (*p* < 0.001).

In the cultivated *P. australis* region, significant correlations were observed between the relative abundance of the most dominant phyla Firmicutes and norfloxacin (NOR) and total antibiotics (SUM) in pore water samples (*p* < 0.001). What is more, the relative abundance of Acidobacteriota also has significant correlations with antibiotics, such as SPD, ciprofloxacin (CIP), oxytetracycline (OTC), and tetracycline (TC), in pore water samples from cultivated *P. australis* region (*p* < 0.001). As for sediments in the cultivated *P. australis* region, the relative abundance of Chloroflex, Acidobacteriota, and Firmicutes showed significant correlations with ORP, WC and DOC, and OTC (*p* < 0.001).

## 5. Discussion

Antibiotic distribution behavior exists between sediment and pore water (Cheng et al., 2014), moreover, the distribution coefficient of antibiotics from sediment to pore water was higher than that from sediment to overlying water (Xu et al., 2014). Therefore, the sediment, as a source of pollutants, is more likely to release antibiotics into pore water, which may lead to a significant difference in bacterial community diversity between pore water and sediment in both wild and cultivated *P. australis* regions. Previous studies have also shown that sediment-associated bacterial communities are more abundant and diverse than bacteria in pore water (Keshri et al., 2018). The aquatic plant root system can influence the connection between pore water and sediments, where the physical, chemical, and biological components interact closely (Li et al., 2019). Plants can rely on beneficial interactions between roots and bacterial communities to obtain nutrients, degrade contaminants, and promote growth (Edwards et al., 2015; Wang et al., 2022), so the interactions might be changed by rich roots in the cultivated *P. australis* region under antibiotic stress.

In the current study, the bacterial community diversity (i.e., Shannon and Shannoneven) in sediments was higher than that in pore water from both *P. australis* regions under the different antibiotics stress, and no significant difference in diversity in sediment was observed between wild and cultivated *P. australis* regions, which can be explained by the clear fact that wild and cultivated *P. australis* all promote the migration of bacterial communities between pore water and sediments. Moreover, there was no significant difference in the bacterial community structure in pore water between the wild and cultivated *P. australis* regions under the same antibiotics stress. The possible explanation was no significant difference in the total antibiotics in pore water between both regions (Zhang et al., 2022a,b), What is more, the similar bacterial community in sediments between both regions might be explained by the fact that the two *P. australis* regions were connected by inflow rivers, and the bacterial communities may coalesced between pore water and sediments (Ren et al., 2017; Gao et al., 2021). It is different from the previous research that the bacterial community in pore water in different types of wetlands can be distinguished (Wang et al., 2018).

Furthermore, the composition of bacterial communities changed under the influence of antibiotics, while Source Tracker analysis results showed that 1.28% variations in sediment bacterial composition could be attributed to the contribution of the pore water in both *P. australis* regions, which can be explained by the clear fact that pore water contains the same levels of antibiotics in both *P. australis* regions. Gao et al. (2021) report that the contribution of the bacterial community from the water to sediment is lower (<0.3%), similarly, little proportion or none from sediments to the water was observed. However, the contribution (2.04%) of the bacterial community from the sediment to pore water in the cultivated *P. australis* region is higher than that (1.01%) in the wild *P. australis* region. Therefore, aquatic plant rich roots might alter the migration of bacterial communities between pore water and sediment. We speculated that plant cultivation might change the source-sink pattern between sediments and pore water. Similar dominant phyla (e.g., Actinobacteriota, Chloroflexi, and Proteobacteria) in sediments and different dominant phyla in pore water were observed in both *P. australis* regions. This might be associated with sediment textures and the hydraulic or zoobenthos disturbances (Zhu et al., 2022b) in both regions (Supplementary Figure S1). Additionally, the root exudates of wild and cultivated *P. australis* and water quality in both regions might be different, which will also affect the bacterial community in the pore water (Chen et al., 2019; Song et al., 2020). Previous studies have reported that the development of bacterial communities in the rhizosphere sediments is related to plant species (Marschner et al., 2004). Therefore, we hypothesized that the difference in bacterial communities in the pore water might be caused by different root systems of wild and cultivated *P. australis* in the current study.

In the current study, in the wild *P. australis* region, NH_4_-N, NO_3_-N, and sediment particle size were significantly associated with the relative abundance of major bacterial phyla in pore water or sediments, while antibiotics (e.g., TC, OTC, and NOR) were associated with the relative abundance of major bacterial phyla in the cultivated *P. australis* region pore water or sediments. TC and OTC are widely used in the treatmentof human and livestock diseases, livestock and poultry breeding (Van Boeckel et al., 2014; Xu et al., 2022), therefore these antibiotics were introduced to cultivated *P. australis* region and further affect the bacterial community (Karkman et al., 2019).

This study shows that under the influence of antibiotics, higher alpha diversity (e.g., Shannoneven and Shannon) of bacterial communities were observed in the sediments than those in the pore water in both *P. australis* regions. Different compositions of bacterial communities in the pore water and similar composition in sediments in both *P. australis* regions. The contribution of the bacterial community in pore water from cultivated *P. australis* region to sediment is higher (2.04%) than that in wild *P. australis* region (1.01%), therefore, plant cultivation might change the source-sink pattern between sediments and pore water. Generally, pore water and sediments bacterial communities in wild and cultivated *P. australis* regions showed significant differences. Therefore, further studies should be carried out to explore bacteria transfer and coalescence in different aquatic plants under antibiotic stress in a shallow lake in the future.

## Data availability statement

The datasets presented in this study can be found in online repositories. The names of the repository/repositories and accession number(s) can be found in the article/Supplementary material.

## Author contributions

LZ: writing—investigation and original draft. JB: funding acquisition and writing—review and editing. YZ, KZ, RX, and MJ: review and editing. ZW, YW, and HL: writing—review and editing. All authors contributed to the article and approved the submitted version.

## Funding

This study was financially supported by Projects of International Cooperation and Exchanges NSFC-ANID Fund (number 51961125201 in China and code NSFC190012 in Chile).

## Conflict of interest

The authors declare that the research was conducted in the absence of any commercial or financial relationships that could be construed as a potential conflict of interest.

## Publisher’s note

All claims expressed in this article are solely those of the authors and do not necessarily represent those of their affiliated organizations, or those of the publisher, the editors and the reviewers. Any product that may be evaluated in this article, or claim that may be made by its manufacturer, is not guaranteed or endorsed by the publisher.
