# Supplementary material for: Antibiotics affected the bacterial community structure and diversity in pore water and sediments with cultivated Phragmites australis in a typical Chinese shallow lake
Source: Front Microbiol. 2023 Mar 14;14:1155526. doi: 10.3389/fmicb.2023.1155526 (PMC10043375; doi:10.3389/fmicb.2023.1155526)
Supplement: Supplementary file 1 [file Data_Sheet_1.doc]

#
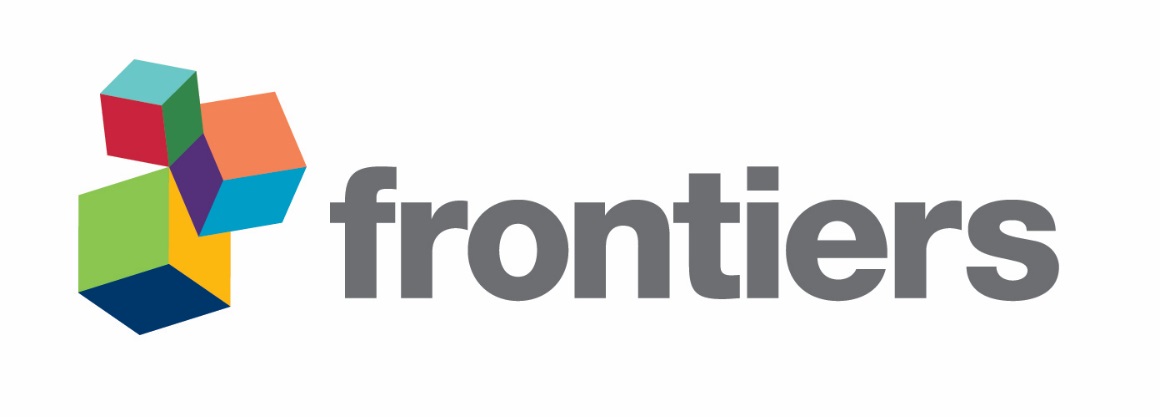


# Supplementary Material

**Antibiotics affected the bacterial community structure and diversity in pore water and sediments with cultivated *Phragmites australis* in a typical Chinese shallow lake**

Ling Zhang^ab^, Junhong Bai*^a^, Yujia Zhai^a^, Kegang Zhang^c^, Zhuoqun Wei^a^, Yaqi Wang^a^, Haizhu Liu^a^, Rong Xiao^d^, Milko A. Jorquera^e^

*a School of Environment, Beijing Normal University, Beijing 100875, China*

*b School of Chemistry and Chemical Engineering, Qinghai Normal University, Xining 810008, China*

*c Department of Environmental Engineering and Science, North China Electric Power University, Baoding, China*

*d College of Environment & Safety Engineering, FuZhou University, Fuzhou, China*

*e Laboratorio de Ecología Microbiana Aplicada (EMALAB), Departamento de Ciencias Químicas y Recursos Naturales, Universidad de La Frontera, Temuco, Chile*

# **Materials and methods**

## 1.1 Sample collection and analysis

To investigate the effects of antibiotics on bacterial communities in pore water and sediments of the cultivated and wild *P. australis* region in Zaozhadian (ZZD) lake，we carried out sampling campaigns in October 2020 (Fig. S1). Sediment samples were collected from 8 representative sampling sites in wild and cultivated *P. australis* in the ZZD Lake. Sampling sites S1 to S4 were in the wild *P. australis* region and S5 to S8 were the cultivated *P. australis* region. Sediment samples were collected by stainless steel static gravity dredger and stored in three parts to determine physicochemical properties, extract DNA for bioinformatics analysis, as well as to extract pore water by centrifugation. All the samples were stored in the freezer and brought back to the laboratory as soon as possible. In the laboratory, one part of the sediments was freeze-dried in a vacuum freeze dryer, ground in an agate mortar, passed through a 0.50 μm sieve and sealed in a plastic bag at - 4 °C before obtaining physicochemical properties. the remaining sediment samples were stored at −80°C to extract DNA. The pore water samples (about 250 mL) were pre-filtered through 0.45 μm membranes to remove solid suspended particulate matter and then filtered through 0.22 μm polytetrafluorethylene (PTFE) filters and stored at -80◦C until DNA extraction. Pore water filtrate through 0.45 μm membrane refrigerated at -4◦C for physicochemical analysis.

Physicochemical parameters of the pore water and sediment samples including pH, EC, water content (WC), dissolved organic carbon (DOC), total phosphorus (TP), total nitrogen (TN, only for water), sediment organic matter (SOM), particle size, dissolved oxygen (DO, only for water), nitrate-nitrogen (NO_3_^−^-N), ammonium-nitrogen (NH_4_^+^-N) were analyzed. These parameters were determined according to the standard methods recommended in our previous research (Lu et al., 2018; Zhang et al., 2023)

Nine target antibiotics were selected including norfloxacin (NOR), ofloxacin (OFL), ciprofloxacin (CIP), oxytetracycline (OTC), tetracycline (TC), sulfapyridine (SPD), sulfapyridine (SDZ), erythromycin (ERM) and roxithromycin (ROM). The detail of nine antibiotics and extraction according to our previous research (Zhang et al., 2023). Briefly, the antibiotics were analyzed by high-performance liquid chromatography (HPLC) mass-spectrometry (MS) (HPLC–MS/MS). An API 4500 QTrap liquid chromatography-mass spectrometer (Applied Biosystems, Foster City, CA, USA) and Waters BEH–C18 column (2.1 mm × 100 mm, particle size 1.7 μm) were used for the determination. The internal standard method was used to quantify antibiotic concentrations. Procedure blanks and solvent blank were set up in each batch of experiments and parallel samples were set up for extraction and analysis to avoid accidental errors. A detailed information can be seen in Zhang et al. (2022).

The total concentration (SUM) of nine antibiotics was quantified for each pore water and sediment sample in our previous studies (Zhang et al., 2022). Briefly, the sediments from cultivated *P. australis* region showed significantly (*p* < 0.05) higher concentrations of sulfapyridine (SPD, 2.48 ± 0.59 ng/L), norfloxacin (NOR, 16.79 ± 0.20 ng/L), ofloxacin (OFL, 30.33 ± 3.42 ng/L), ciprofloxacin (CIP, 10.34 ± 3.25 ng/L) and total concentration (SUM, 128.09 ± 30.20 ng/L) than those in sediments from wild *P. australis* region. The concentration of SPD (150.37 ± 63.18 ng/L), NOR (756.46 ± 331.89 ng/L), OFL (66.96 ± 6.9 ng/L, ), CIP (23.30 ± 4.43 ng/L) and ERM (15.51 ± 2.68 ng/L) were obtained in pore water from cultivated *P. australis* region. However, no significant differences in antibiotic concentrations in pore water were observed between the wild and cultivated *P. australis* region (*p* > 0.05).

## 1.2 DNA extraction and Illumina sequencing

DNA was extracted according to the method reported by Zhang et al. (2022), with some modifications. In brief, around 0.5 g sediment samples and 0.22 μm filtered films for the pore water were subjected to DNA extraction using FastDNA SPIN Kit for Soil (MP Biomedicals, Solon, OH, USA) following the instruction manual. The quality of DNA was checked by spectrophotometric analysis using NanoDrop 2000 UV-vis spectrophotometer (Thermo Scientific, Wilmington, USA). The DNA was stored at −20 °C until use.

Polymerase chain reaction (PCR) amplification was performed using the universal primers 338F (ACTCCTACGGGAGGCAGCAG) and 806R (GGACTACHVGGGTWTCTAAT) designed against the V3-V4 region of bacterial 16S rRNA gene in an ABI GeneAmp® 9700 PCR thermocycler (ABI, CA, USA). Amplicon sequencing was performed using the Illumina MiSeq PE300 platform at the Shanghai Majorbio Bio-pharm Technology Co., Ltd, in China. The PCR amplification of 16S rRNA gene was performed, initial denaturation at 95 °C for 3 mins, followed by 30 cycles of denaturing at 95 °C for 30 s, annealing at 55 °C for 30 s and extension at 72 °C for 45 s, and single extension at 72 °C for 10 min. The details of DNA amplification, Illumina sequencing, and raw sequences processing can be seen in Zhang et al.(2022). Amplicon sequence variants (ASVs) were obtained and representative sequence and abundance information for bioinformatics analysis. Alpha diversities were used in our study to evaluate bacterial community richness (i.e., Sobs), diversity (Shannon), evenness (Shannoneven), and Phylogenetic diversity (PD). The sequence data were submitted to NCBI Sequence Read Archive with the accession number PRJNA929306.

## 1.3 Statistical analysis

Alpha diversity was calculated at the ASV levels. One-way ANOVA analysis was performed to test the differences in the alpha diversity between the pore water and sediment samples, as well as species differences between groups. The significance level was described as *p* < 0.05, *p* < 0.01, or *p* < 0.001. Spearman’s correlation was conducted to identify the correlations between the bacteria phyla and environmental factors. Beta diversity is expressed as principal coordinate analysis (PCoA), which both were performed on the free online platform of Majorbio Cloud Platform (https://cloud.majorbio.com/) (Yu et al., 2022). Statistical analysis was performed using SPSS 24.0 for Windows or in R (v4.1.1, <https://www.r-project.org/>). Source Tracker was applied to estimate the relative contributions of the pore water and sediment samples that originated from each other (Sun et al., 2022).

# **2、Figures**


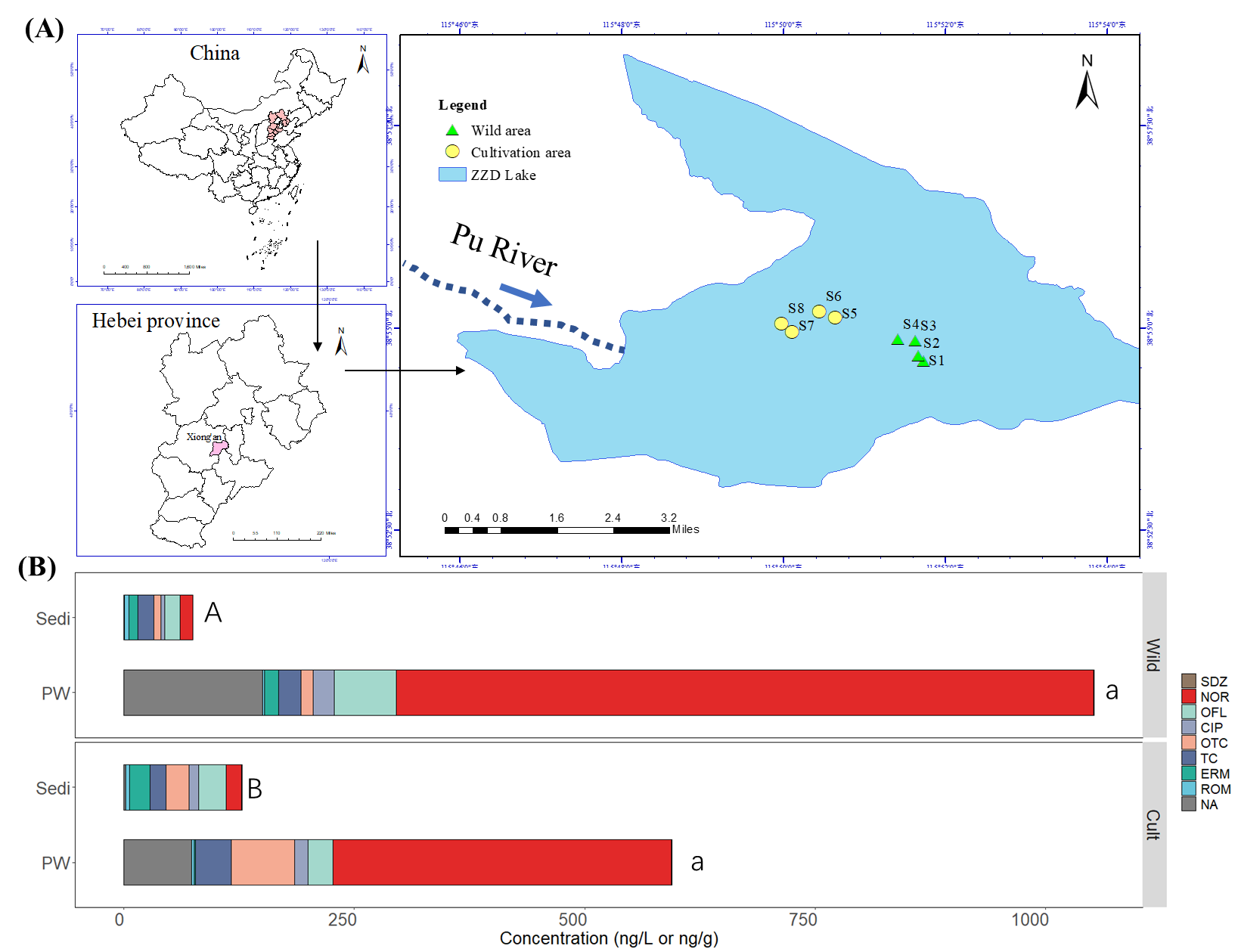


### Fig. S1 Location map of sampling sites in the Baiyangdian (BYD) lake (A) and distribution of nine antibiotics in pore water and sediments in wild and cultivated *P. australis* regions (Zhang et al., 2022). SPD: sulfapyridine, SDZ: sulfapyridine, NOR: norfloxacin, OFL: ofloxacin, CIP: ciprofloxacin, OTC: oxytetracycline, TC: tetracycline, ERM: erythromycin, ROM: roxithromycin, PW: pore water, Sedi: sediments. Different letters represent significant differences in pore water or sediments antibiotics levels between wild and cultivated *P. australis* regions.

### Fig. S2. Variations in bacterial alpha and beta diversity (A) and principal coordinates analysis (PCoA) (B) on ASV level in the pore water (PW) and sediments (Sedi) from the wild and cultivated P. australis regions.


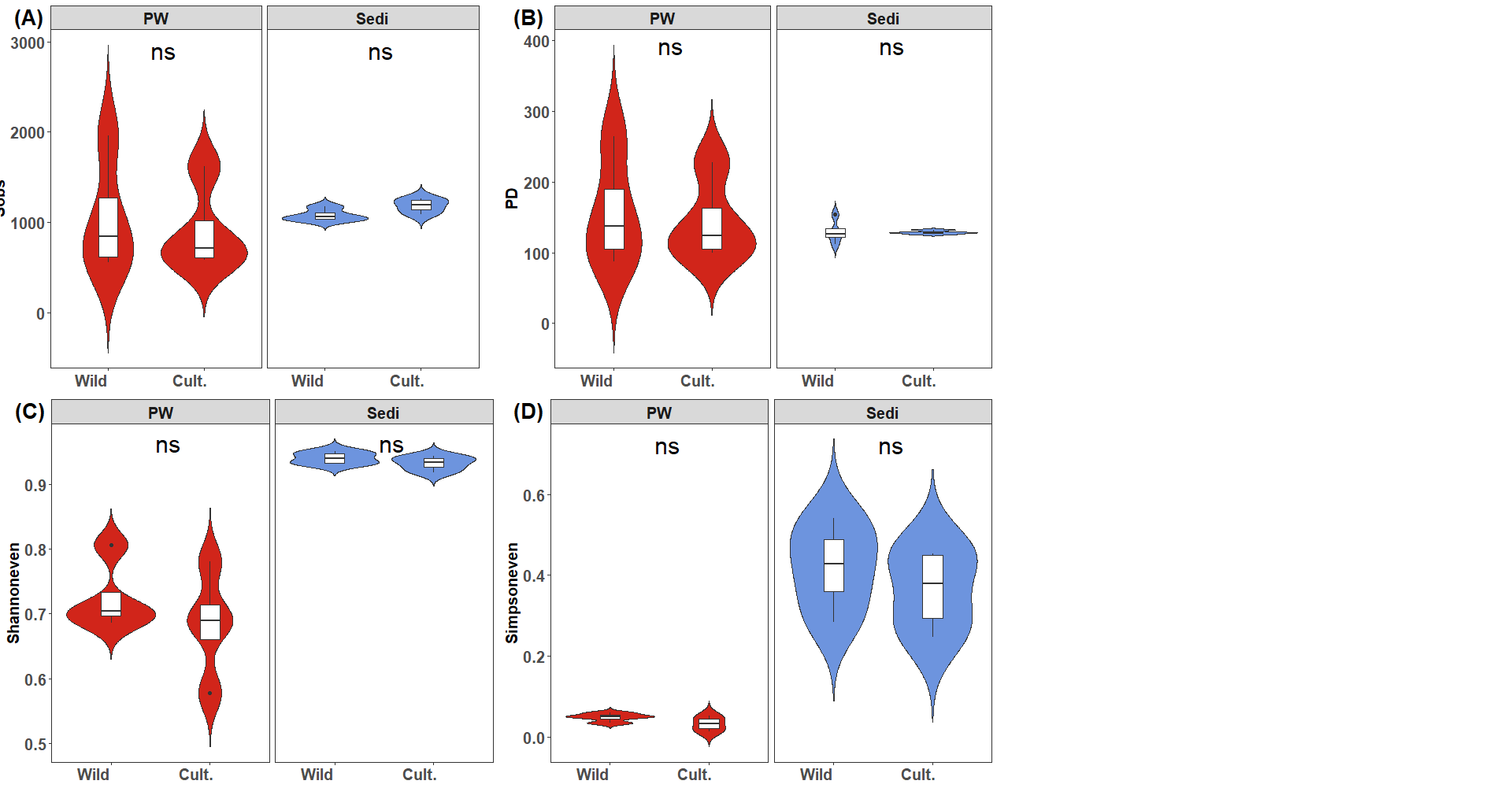


### Fig. S3. Variations in bacterial alpha and beta diversity between wild and cultivated P. australis region

### Fig. S4. phyla difference analysis in all bacterial alpha in two group

# **References**

Lu, Q., Bai, J., Zhang, G., Zhao, Q., Wu, J., 2018. Spatial and seasonal distribution of carbon, nitrogen, phosphorus, and sulfur and their ecological stoichiometry in wetland soils along a water and salt gradient in the Yellow River delta, China. Physics and chemistry of the earth. Parts A/B/C. 104, 9-17. DOI:10.1016/j.pce.2018.04.001

Sun, C., Xiong, W., Zhang, W., Liu, Z., Li, Y., Zhou, X., Niu, L., Zhang, H., Wang, L., 2022. New insights into identifying sediment phosphorus sources in river-lake coupled system: A framework for optimizing microbial community fingerprints. Environmental Research. 209, 112854. DOI:10.1016/j.envres.2022.112854

Yu, L., Bai, J., Huang, L., Zhang, G., Wang, W., Wang, X., Yu, Z., 2022. Carbon-rich substrates altered microbial communities with indication of carbon metabolism functional shifting in a degraded salt marsh of the Yellow River Delta, China. Journal of Cleaner Production. 331, 129898. DOI:10.1016/j.jclepro.2021.129898

Zhang, L., Bai, J., Wang, C., Wei, Z., Wang, Y., Zhang, K., Xiao, R., Jorquera, M.A., Acuña, J.J., Campos, M., 2022. Fate and ecological risks of antibiotics in water-sediment systems with cultivated and wild Phragmites australis in a typical Chinese shallow lake. Chemosphere. 305, 135370. DOI:10.1016/j.chemosphere.2022.135370

Zhang, L., Bai, J., Zhang, K., Wang, Y., Xiao, R., Campos, M., Acuña, J., Jorquera, M.A., 2023. Occurrence, bioaccumulation and ecological risks of antibiotics in the water-plant-sediment systems in different functional areas of the largest shallow lake in North China: Impacts of river input and historical agricultural activities. The Science of the total environment. 857, 159260. DOI:10.1016/j.scitotenv.2022.159260
